# Bullous pemphigoid anti-BP180-NC16A autoantibody reactivity in healthy individuals is associated with marked hypovitaminosis D and Th2-like cytokine predominance

**DOI:** 10.1007/s00403-022-02386-4

**Published:** 2022-08-12

**Authors:** Stefan Tukaj, Katja Bieber, Wiebke Prüßmann, Jasper N. Prüßmann, Enno Schmidt, Detlef Zillikens, Ralf J. Ludwig, Michael Kasperkiewicz

**Affiliations:** 1 Department of Molecular Biology, Faculty of Biology, University of Gdańsk, Wita Stwosza 59, 80-308 Gdańsk, Poland; 2 Lübeck Institute of Experimental Dermatology and Center for Research On Inflammation of the Skin, University of Lübeck, Lübeck, Germany; 3 Department of Dermatology and Center for Research On Inflammation of the Skin, University of Lübeck, Lübeck, Germany; 4 Department of Dermatology, Keck School of Medicine, University of Southern California, Los Angeles, CA USA

**Keywords:** Bullous pemphigoid, Vitamin D, 25(OH)D, Th2, Cytokine

## Abstract

Autoimmune bullous disease autoantibodies, particularly including bullous pemphigoid (BP)-related anti-BP180-NC16A IgG, have been reported in a small subset of healthy individuals, but information about associated factors is lacking. We hypothesized that an abnormal status of immunomodulatory vitamin D could play a role in anti-BP180-NC16A autoantibody reactivity in healthy persons. In addition, we aimed to evaluate the cytokine profile associated with these autoantibodies. Plasma samples from 34 anti-BP180-NC16A IgG-reactive and 85 anti-BP180-NC16A IgG-negative healthy blood donors were tested for levels of 25-hydroxyvitamin D [25(OH)D] and a wide range of cytokines (IL-2, IL-4, IL-5, IL-6, IL-9, IL-10, IL-13, IL-17A, IL-17F, IL-21, IL-22, IFN-γ, and TNF-α). We observed that anti-BP180-NC16A IgG-reactive healthy subjects had significantly lower plasma 25(OH)D levels and about a two-fold higher rate of vitamin D deficiency (< 20 ng/ml) compared to anti-BP180-NC16A IgG-negative healthy persons. In addition, anti-BP180-NC16A IgG-positive samples were characterized by significantly higher levels of IL-2, IL-5, IL-9, IL-10, and IL-13 which were, however, not significantly associated with the vitamin D levels. Our results indicate that healthy individuals with BP autoantibody reactivity share similarities with BP patients regarding the vitamin D status and cytokine profile (i.e., marked hypovitaminosis D and Th2 predominance), which may have pathophysiologic implications.

## Introduction

In up to 10% of healthy individuals, circulating autoantibodies, such as antinuclear antibodies (ANA) and autoantibodies directed against desmosomal and hemi-desmosomal structural proteins, are detected [1–4]. These autoantibodies, which are characteristic of rheumatic diseases and autoimmune bullous diseases (AIBD), respectively, have been described to be present for up to several years before some individuals eventually become symptomatic [3, 4].

In a large population of more than 7000 normal blood donors studied for pemphigus and pemphigoid autoantibodies, we previously showed that the cumulative prevalence of these autoantibodies was 0.9%, with bullous pemphigoid (BP)-associated anti-BP180-NC16A IgG being most prevalent (0.5%) [2]. Based on incidences of BP, the by far most common AIBD in Central Europe and Northern America [5], it has been hypothesized that approximately every 37th healthy individual with anti-BP180-NC16A IgG reactivity may develop BP [2]. However, information about factors associated with such autoantibodies in healthy individuals, including biomarkers distinguishing between resistant antibody-positive individuals and those who are susceptible to develop clinical disease, is lacking.

Similar to what has been previously described in ANA-positive healthy individuals [6, 7], we hypothesized that an abnormal status of immunomodulatory vitamin D could play a role in anti-BP180-NC16A reactivity in healthy people. In fact, hypovitaminosis D has been reported to be more common in AIBD patients than in the general population [8, 9], but nothing is known about vitamin D and BP autoantibodies in healthy populations. In addition, we aimed to evaluate the cytokine profile of anti-BP180-NC16A IgG-reactive versus anti-BP180-NC16A IgG-negative healthy blood donors, considering that an altered cytokine expression is found in BP patients [5].

## Materials and methods

### Blood samples

This investigation included 119 anonymized plasma samples of our previously described study cohort of normal blood donors from the Institutes for Transfusion Medicine Lübeck, Kiel, and Frankfurt (Germany) [2]. 34 anti-BP180-NC16A IgG-reactive and 85 anti-BP180-NC16A IgG-negative specimens were used. Anti-BP180-NC16A IgG was detected by both the biochip-based indirect immunofluorescence assay (dermatology-mosaic 7) and the anti-BP180-NC16A enzyme-linked immunosorbent assay (ELISA; all from EUROIMMUN AG, Lübeck, Germany) [2]. Use of human biological material was approved by the ethics committee of the University of Lübeck, and written informed consents were obtained in accordance with the Declaration of Helsinki.

### Vitamin D measurement

Plasma 25-hydroxyvitamin D [25(OH)D] levels were measured by a 25(OH) Vitamin D ELISA Kit (Enzo Life Sciences, Lörrach, Germany) according to the manufacturer’s instructions.

### Multiplex cytokine assay

Plasma concentrations of various cytokines (IL-2, IL-4, IL-5, IL-6, IL-9, IL-10, IL-13, IL-17A, IL-17F, IL-21, IL-22, IFN-γ, and TNF-α) were determined by the LEGENDplex human Th cytokine panel (13-plex) array (Biolegend, Fell, Germany) according to the manufacturer’s protocol. The analysis was performed with the flow cytometer MACSQuant Analyzer 10 (Miltenyi Biotec, Bergisch Gladbach, Germany).

### Statistical analysis

Statistical calculations were performed using GraphPad Prism software (GraphPad, San Diego, CA, USA). Non-normal and normal distributed data were analyzed by Mann–Whitney *U* test and Welch’s *t*-test, respectively. In addition, Spearman’s rank correlation test was used. *P* values less than 0.05 were considered as significant.

## Results

### Anti-BP180-NC16A IgG-positive healthy individuals have markedly low vitamin D levels

**Fig. 1:**
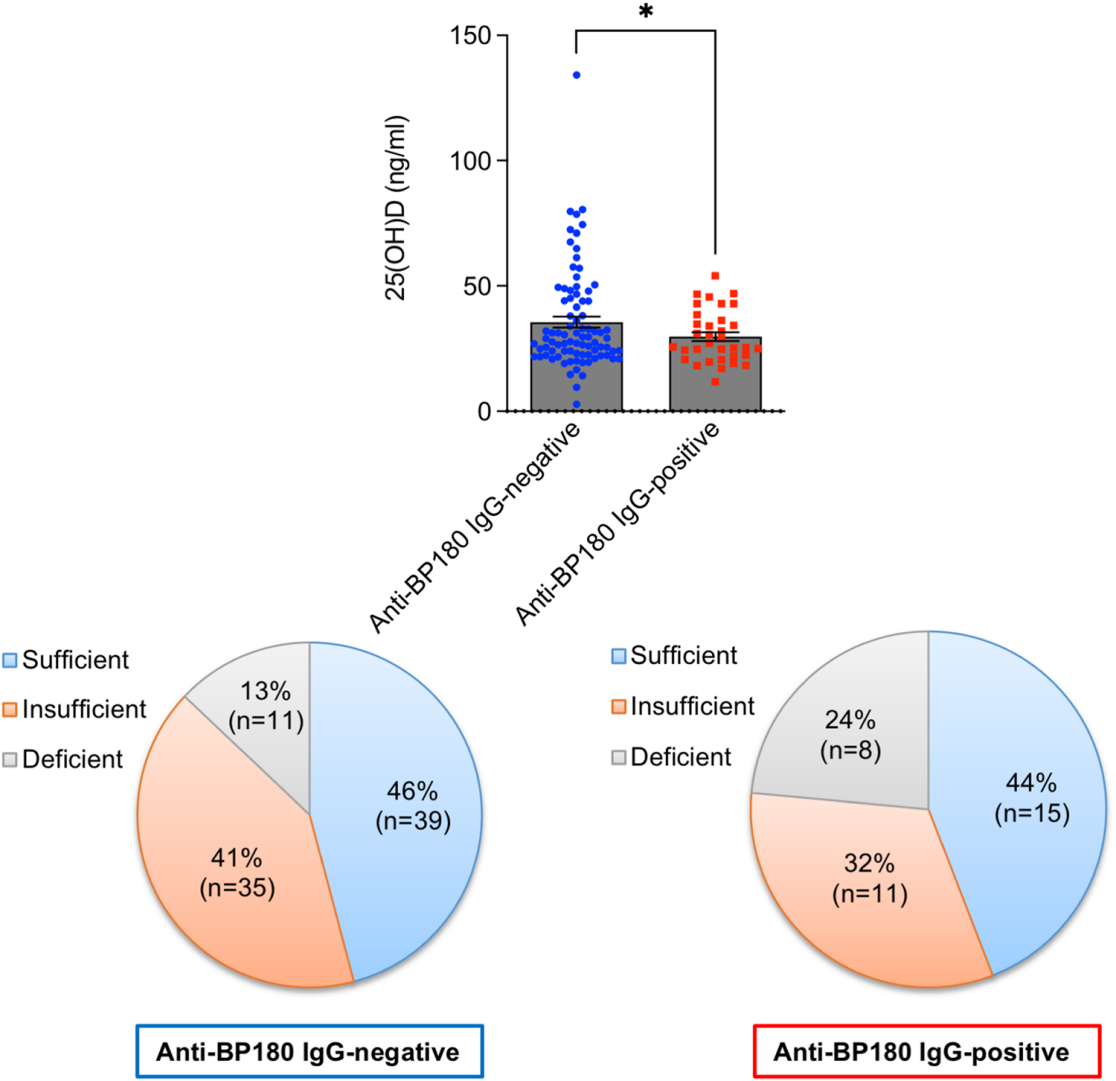
Anti-BP180 autoantibody reactivity in healthy individuals is associated with marked hypovitaminosis D. Plasma 25-hydroxyvitamin D [25(OH)D] status of anti-BP180-NC16A IgG-positive (*n* = 34) and anti-BP180-NC16A IgG-negative (*n* = 85) healthy individuals measured by enzyme-linked immunosorbent assay. 25(OH)D plasma levels of ≥ 30 ng/ml, 20–29 ng/ml, and < 20 ng/ml were considered sufficient, insufficient, and deficient, respectively. The squares/dots and bars indicate individual and mean (± SEM) values in each group, respectively. *P < 0.05

We found that individuals with anti-BP180-NC16A autoantibody reactivity had significantly lower 25(OH)D plasma levels as compared to anti-BP180-NC16A IgG-negative people. In addition, while a state of hypovitaminosis D was found in the majority of analyzed healthy individuals irrespective of anti-BP180-NC16A autoantibody reactivity, the rate of vitamin D deficiency, defined as < 20 ng/ml according to the Endocrine Society [10], was approximately twice as high in anti-BP180-NC16A IgG-positive compared to anti-BP180-NC16A IgG-negative healthy individuals (24% vs. 13%, respectively) (Fig. 1).

### A Th2-dominant milieu is found in anti-BP180-NC16A IgG-positive healthy individuals

**Fig. 2:**
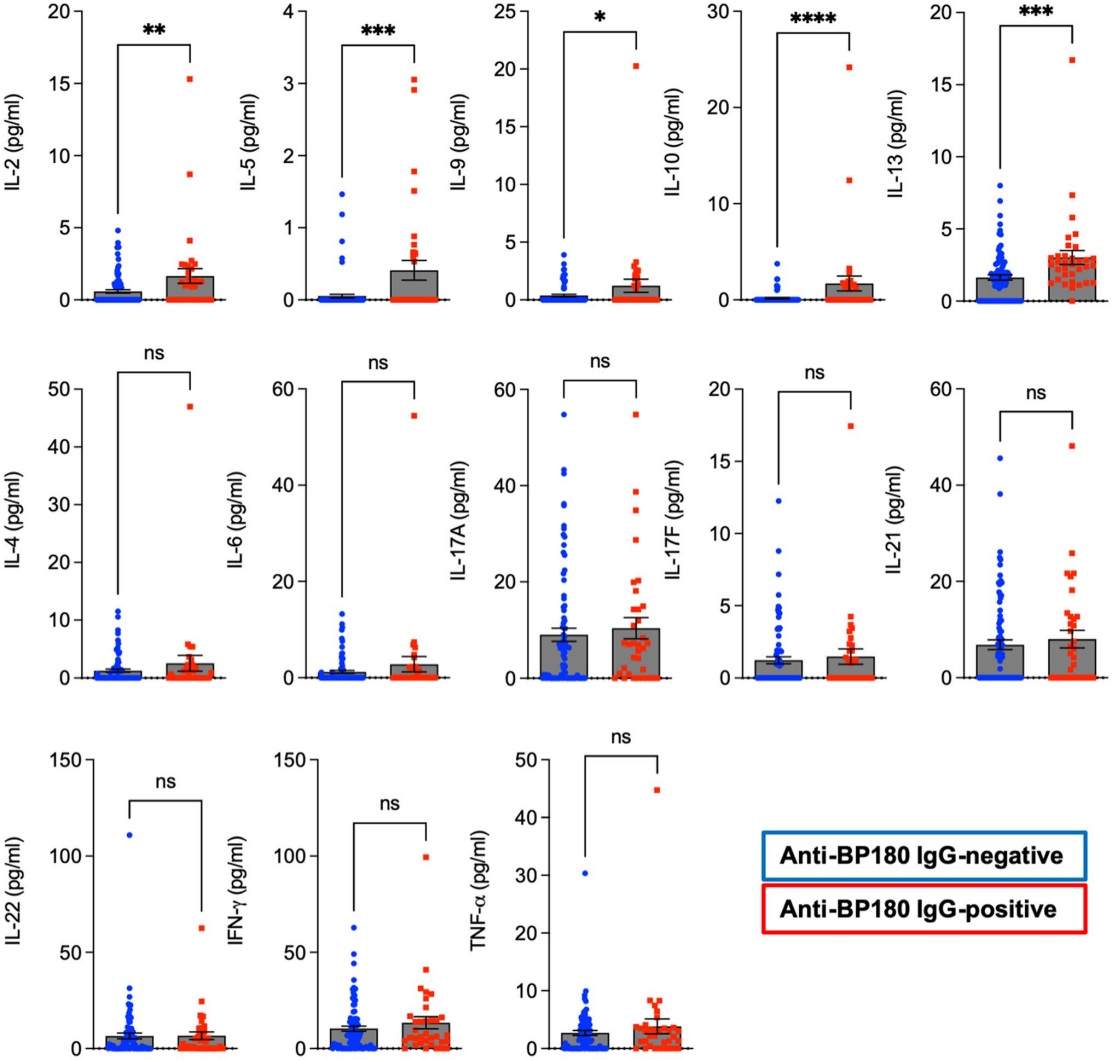
Anti-BP180 autoantibody reactivity in healthy individuals is associated with Th2 predominance. Plasma levels of IL-2, IL-4, IL-5, IL-6, IL-9, IL-10, IL-13, IL-17A, IL-17F, IL-21, IL-22, IFN-γ, and TNF-α in anti-BP180-NC16A IgG-positive (*n* = 34) and anti-BP180-NC16A IgG-negative (*n* = 85) healthy individuals measured by flow cytometry. The squares/dots and bars indicate individual and mean (± SEM) values in each group, respectively. *P < 0.05, **P < 0.01, ***P < 0.001, ****P < 0.0001. *ns* not significant

We observed that, in comparison to anti-BP180-NC16A IgG-negative individuals, anti-BP180-NC16A IgG-positive ones were characterized by significantly higher plasma levels of IL-2, IL-5, IL-9, IL-10, and IL-13 (Fig. 2). Of note, these cytokines, except IL-2, represent Th2-based immune responses.

### Vitamin D status is not correlated with the Th2-dominant milieu in anti-BP180-NC16A IgG-positive healthy individuals

**Fig. 3:**
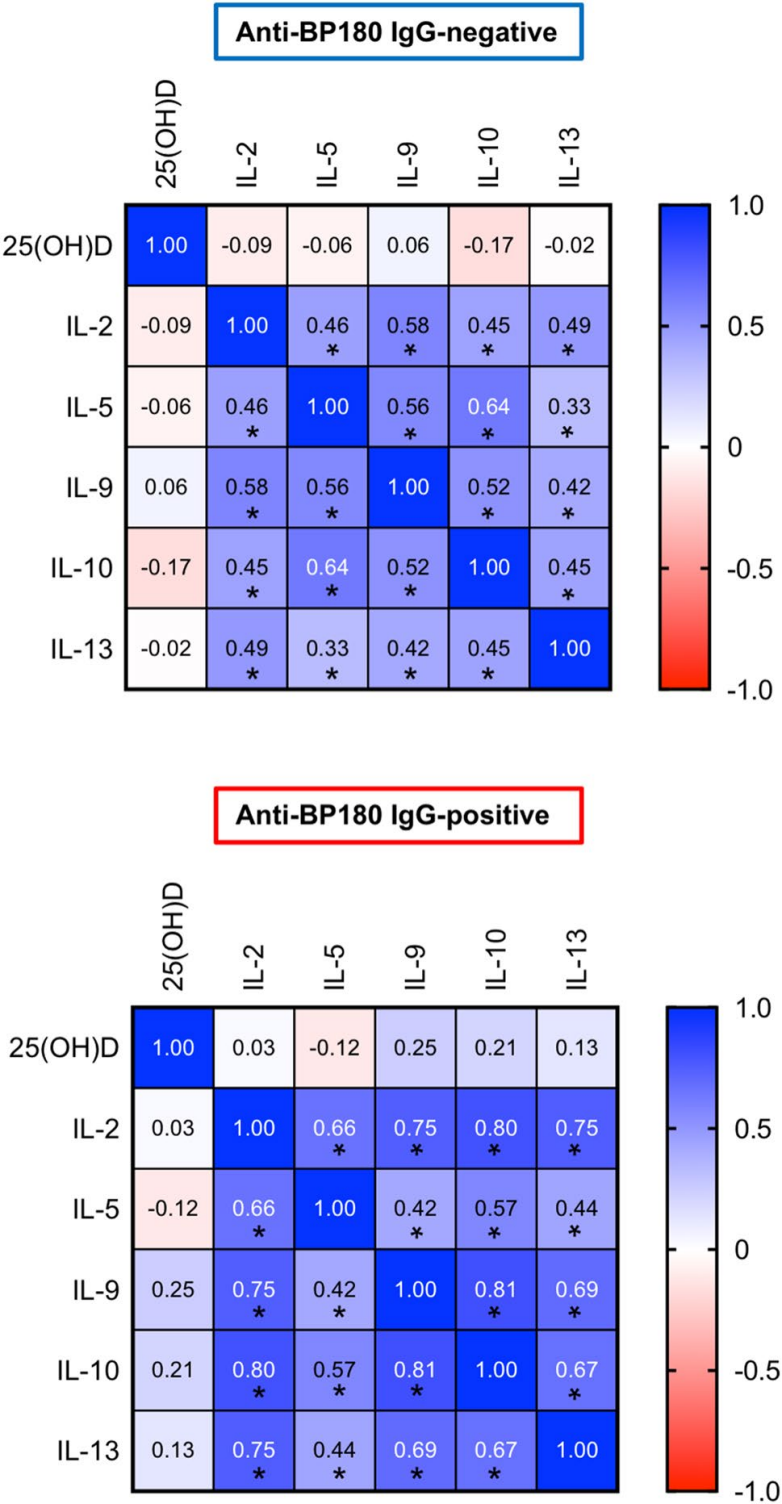
Hypovitaminosis D is not correlated with the Th2-dominant milieu in anti-BP180-NC16A IgG-positive healthy subjects. Heatmap matrix plot of Spearman’s rank correlation coefficients (*r*) between plasma levels of 25(OH)D, IL-2, IL-5, IL-9, IL-10, and IL-13 in either anti-BP180-NC16A IgG-negative or anti-BP180-NC16A IgG-positive healthy individuals. *R* values are presented in boxes and significant correlations are indicated by asterisks. *P < 0.05

25(OH)D levels were not significantly correlated with levels of IL-2, IL-5, IL-9, IL-10, or IL-13 in neither anti-BP180-NC16A IgG-negative nor anti-BP180-NC16A IgG-positive healthy individuals, although positive mutual correlations between the analyzed cytokines were recorded irrespective of anti-BP180-NC16A autoantibody positivity (Fig. 3).

## Discussion

The hormonally active, UVB-induced vitamin D metabolite, 1,25-dihydroxyvitamin D_3_ (calcitriol), participates in regulation of immune responses through binding to the vitamin D receptor expressed in multiple cells of the immune system [8]. There are several lines of evidence indicating vitamin D involvement in AIBD. A meta-analysis of 9 case–control studies revealed that the vitamin D level of patients with AIBD including BP was significantly lower than that in controls [9]. On an experimental level, we previously showed that calcitriol inhibited BP180-NC16A IgG-induced secretion of pro-inflammatory cytokines in a human keratinocyte culture [11]. In addition, calcitriol promoted regulatory T and B cells, inhibited pro-inflammatory Th17 cells and neutrophils, and attenuated autoantibody production in a mouse model of the BP-like pemphigoid disease epidermolysis bullosa acquisita [12]. Moreover, previous studies observed that vitamin D inhibited the expression of pemphigus and pemphigoid autoantigens [13, 14]. In line with these reports, our current work showed that anti-BP180-NC16A IgG-reactive healthy individuals have significantly lower plasma vitamin D levels and about a two-fold higher percentage of vitamin D deficiency compared to anti-BP180-NC16A IgG-negative healthy persons. These findings are intriguing in two ways, as previously discussed in a similar context of ANA-positive healthy individuals [6]. First, the observation that both BP autoantibody-positive healthy individuals and BP patients have reduced vitamin D indicates that the molecular mechanisms by which hypovitaminosis D would predispose to autoimmunity operate early in BP development, prior to the possible onset of clinical symptoms. Second, considering that AIBD patients possess some risk factors for hypovitaminosis D (i.e., skin lesions and pain may limit outdoor activities, where sun exposure may in turn trigger the disease), whereas BP180-NC16A IgG-positive healthy individuals do not [9], it can be therefore assumed that an abnormal vitamin D status is not solely the result of disease-associated lifestyle changes. Nevertheless, it needs to be mentioned that more than half of anti-BP180-NC16A IgG-negative healthy individuals also had inadequate plasma vitamin D levels, confirming that hypovitaminosis D is common in the general population (irrespective of subclinical autoimmune disease phenomena) [9, 15, 16].

A potential pathogenic relevance of the detected anti-BP180-NC16A autoantibodies in healthy individuals has been proposed [2]. We could previously show that these autoantibodies formed immune complexes with recombinant antigen and dose-dependently stimulated neutrophils in vitro, although fine-epitope mapping within NC16A revealed differences in the binding pattern between healthy individuals and BP patients [2]. BP is considered a Th2-mediated disease in which the presence of autoreactive T cells with increased, therapeutically relevant Th2-associated cytokines (e.g., IL-5, L-10, and IL-13) in serum and blister fluid has been reported [5, 17]. Our observation that these Th2 cytokines were predominantly elevated in the blood of anti-BP180-NC16A IgG-reactive compared to anti-BP180-NC16A IgG-negative individuals further suggests a subclinical, BP-like pathogenic environment which may potentially turn into full-blown disease by additional yet-to-be-defined factors.

In contrast, we did not find a direct correlation between the low vitamin D status and the upregulated Th2 cytokines in anti-BP180-NC16A IgG-reactive individuals, which may at least partly be explained by the fact that we analyzed normal blood donors without any known immunological diseases. In fact, different studies indicated variable correlations between vitamin D levels and the cytokine output by Th2 cells, with mostly allergic disorders showing an inverse relationship while some autoimmune diseases (e.g., systemic lupus erythematosus) or healthy subjects were lacking associations [18–20]. Interestingly, we observed a series of positive cross-correlations between the studied cytokines (i.e., IL-2, IL-5, IL-9, IL-10, and IL-13). The presence of these associations may either suggest a physiological interplay or a convergent pathway toward Th2 polarization characteristic of the autoimmune process in BP patients.

Limitations of this study include lack of information regarding demographic data and long-term follow-up of the blood donors.

## Conclusion

In conclusion, our results indicate that healthy individuals with BP autoantibody reactivity share similarities with BP patients regarding the vitamin D status and cytokine profile (i.e., marked hypovitaminosis D and Th2 predominance), which may have pathophysiologic implications.
